# A New Remedy for Burns

**Published:** 1887-07

**Authors:** W. C. Wile


### A NEW REMEDY FOR BURNS.

By W. C. Wile, M. D.

Many remedies have at one time or another been proposed for the surgical condition following the application of excessive heat to the body, and, while some of these are of value, still all are more or less unsatisfactory. The alleviation of the pain and suffering attendant upon burns is one of the most important points in the case, toward which the surgeon directs his efforts. The shock from this cause alone is sufficient, oftentimes, to produce death, and always is great. Accidentally, I recently discovered a remedy, which is easily applied and exceedingly prompt in its action. I was called in some haste to a little child, about three weeks ago, who was badly burned about the hands and face from falling on a hot stove. The burns were deep, the pain excessive, and the shock very considerable. I hastily sent to the drug store for a mixture of lime water, olive oil and carbolic acid. While waiting for this, I prepared to give the child a hypodermic injection of morphine, with which to allay the agony, which was so great that convulsions seemed imminent. While I was getting ready to do this, I espied upon the shelf a bottle of pinus canadensis (colorless), which I had some time before ordered to be diluted as a vaginal wash for the mother. Remembering its wonderful soothing influence in acute inflammations of the vagina, I at once concluded to try it. Taking a corner of a soft handkerchief, I rapidly painted the injured parts, when, like magic, the pain ceased. You can well imagine my surprise and delight at the result. I directed a camel's hair brush to be purchased, and had the mother make free applications, and the case had no other treatment, save a little iodoform ointment later on. Since this, I have tried it in several cases, both slight and severe, and with the same delightful results. The preparation used was made by the Rio Chemical Co., of St. Louis, Missouri.—*Medical Register, Philadelphia, May* 11, 1887.

